# Prenatal origins of suicide mortality: A prospective cohort study in the United States

**DOI:** 10.1038/s41398-021-01777-x

**Published:** 2022-01-10

**Authors:** Pablo Vidal-Ribas, Theemeshni Govender, Rajeshwari Sundaram, Roy H. Perlis, Stephen E. Gilman

**Affiliations:** 1 Social and Behavioral Sciences Branch, Division of Population Health Research, Eunice Kennedy Shriver National Institute of Child Health and Human Development, National Institutes of Health, Bethesda, MD USA; 2 Biostatistics and Bioinformatics Branch, Division of Population Health Research, Eunice Kennedy Shriver National Institute of Child Health and Human Development, National Institutes of Health, Bethesda, MD USA; 3 Center for Quantitative Health, Center for Genomic Medicine and Department of Psychiatry, Massachusetts General Hospital, Boston, MA USA; 4 Department of Psychiatry, Harvard Medical School, Boston, MA USA; 5 Department of Mental Health, Johns Hopkins Bloomberg School of Public Health, Baltimore, MD USA

**Keywords:** Predictive markers, Psychiatric disorders

## Abstract

Most suicide research focuses on acute precipitants and is conducted in high-risk populations. Yet, vulnerability to suicide is likely established years prior to its occurrence. In this study, we aimed to investigate the risk of suicide mortality conferred by prenatal sociodemographic and pregnancy-related factors. Offspring of participants (*N* = 49,853) of the Collaborative Perinatal Project, a U.S. population-based cohort of pregnancies enrolled between 1959 and 1966, were linked to the U.S. National Death Index to determine their vital status by the end 2016. We examined associations between sociodemographic factors during pregnancy, pregnancy complications, labor and delivery complications, and neonatal complications with suicide death coded according to ICD-9/10 criteria. By the end of 2016, 3,555 participants had died. Of these, 288 (214 males, 74 females) died by suicide (incidence rate = 15.6 per 100,000 person-years, 95% Confidence Interval [CI] = 13.9–17.5). In adjusted models, male sex (Hazard Ratio [HR] = 2.98, CI: 2.26–3.93), White race (HR = 2.14, CI = 1.63–2.83), low parental education (HR = 2.23, CI = 1.38–3.62), manual parental occupation (HR = 1.38, CI = 1.05–1.82), being a younger sibling (HR = 1.52, CI = 1.10–2.11), higher rates of pregnancy complications (HR = 2.36, CI = 1.08–5.16), and smoking during pregnancy (HR = 1,28, CI = 0.99–1.66) were independently associated with suicide risk, whereas birth and neonatal complications were not. Consistent with the developmental origins of psychiatric disorders, vulnerability to suicide mortality is established early in development. Both sociodemographic and pregnancy factors play a role in this risk, which underscores the importance of considering life course approaches to suicide prevention, possibly including provision of high-quality prenatal care, and alleviating the socioeconomic burdens of mothers and families.

## Introduction

Suicide thoughts and behaviors increase dramatically after puberty [1, 2], followed by an increase of suicide rates during late adolescence and early adulthood [3], highlighting the need of early risk identification. Most research on suicide, though, emphasizes proximal or acute risk factors among high-risk individuals [4] despite suicide being a multicausal phenomenon influenced by distal and proximal risk factors acting at multiple levels and stages in life [5–7]. Vulnerability to suicide is likely established early in life as it happens with the vulnerability to psychiatric conditions, which partly originates during fetal development, and are one of the major risk factors of suicide. Prenatal and perinatal conditions leading to fetal undernutrition, along with psychological distress, substance use during pregnancy, maternal and paternal age, and parental sociodemographic factors can impact offspring neurodevelopment and lead to higher risk for depression [8, 9], psychosis [10], disruptive behavior disorders [11, 12], and other mental health problems [13, 14].

Findings from a recent meta-analysis of prospective studies suggested that the same applies to suicide risk: sociodemographic (e.g., older birth order, young mother age, single marital status, low parental education, pooled OR range 1.36–1.80) and pregnancy-related factors (e.g., low birth weight, pooled OR range 1.18–1.30) are also associated with suicide mortality in the offspring [15]. However, among previous studies, mostly conducted in European countries, very few have examined both sociodemographic and pregnancy-related factors simultaneously, and in those studies that tested both, the number of risk factors examined has been limited [15]. Since sociodemographic circumstances are associated with prenatal care and pregnancy outcomes [16, 17], examining whether sociodemographic and pregnancy-related factors have independent associations with suicide death in offspring can enhance our understanding of the early life origins of suicide risk and ultimately lead to greater precision in the development of targeted interventions. This meta-analysis also highlighted that few studies have considered family history of psychiatric problems as a confounder in the association between early risk factors and suicide mortality. Likewise, only over a dozen of studies have examined sex differences in such associations, with half of them reporting differences [18–23]. However, results are inconsistent (e.g., lower socioeconomic status linked to suicide in only men [19] or female offspring [23]) and limited by the low number of suicide cases in females.

To this aim, we conducted a data linkage study between the largest pregnancy cohort in the United States (US), the Collaborative Perinatal Project (CPP) cohort [24], and the US National Death Index (NDI) to examine whether prenatal and perinatal factors, including sociodemographic and pregnancy-related factors, were independently associated with increased vulnerability to suicide mortality in offspring followed to middle adulthood. The CPP was established to study prenatal and obstetric antecedents of children’s neurological disorders; as a result, it included a thorough assessment of sociodemographic, pregnancy, birth, and neonatal factors, including maternal psychiatric history. Delivery and neonatal complications have been examined in very few studies in relation to suicide death. However, a recent study examining a wide range of early risk factors found that pregnancy, but not delivery and neonatal complications, were associated with suicide attempt in young people [25]. Whereas suicide death and suicide attempt are closely related phenomena with many shared risk factors, several proximal risk factors for suicide are particularly relevant for suicide mortality (e.g., lethality of method); in contrast, early life factors that increase the risk for suicide are likely to be more general [15, 26]. Therefore, we hypothesize that early life risk factors previously found to be associated with suicide attempt will also be associated with the risk of suicide mortality through late adulthood [15]. Finally, we also explore sex differences in the conferred risk by early life factors to suicide mortality.

## Subjects and methods

### Participants

The CPP enrolled over 59,000 pregnancies between 1959 and 1966 at 12 academic centers and followed offspring through the first 7 years of life [24]. Participants in the current study included CPP offspring known to be alive at the conclusion of the study and whose identifying information could be abstracted from study records (*n* = 49,853) (see flowchart in Supplemental Fig. S1). The vital status of these participants through December 31st, 2016, was determined by probabilistic linkage to death certificates recorded in NDI. Informed consent was obtained from participants in the CPP. Ethical approval for this study was obtained from the NICHD IRB.

### Suicide mortality

The NDI linkage compared the identifying information of study participants (first name, middle initial, last name, father’s surname for females, birth day, birth month, birth year, sex, race, and state of birth) to identifying information on US death certificates recorded between 1979 and 2016 [27]. All possible matches between study records and death certificates were assigned a probabilistic score which quantified the likelihood that the study record and death certificate belonged to the same person; for study records matching to more than one death certificate, we retained the death certificate with the highest score [28, 29]. Matches with scores above NDI’s validated cutoff point were considered true matches and consequently, the study participant was categorized as deceased. The cutoff points were determined by the NDI on the basis of calibration samples that optimize the sensitivity and specificity of matches in samples with known vital status [27]. Validation studies of linkages to the NDI show a high sensitivity (80 to 92% across studies) and specificity (92%), even when social security numbers were not available [30–32].

For death certificates recorded between 1979 and 1996, we counted as a suicide those death certificates with the following ICD-9 codes listed as the underlying cause of death: E950-E959 (intentional self-harm) and E980-E989 (injury of undetermined intent). For deaths between 1997 and 2016, we counted as suicide death certificates with the following ICD-10 codes: X60–84 (intentional self-harm), Y10–34 (injury of undetermined intent), Y87.0 (sequelae of intentional self-harm), and Y87.2 (sequelae of events of undetermined intent). As many previous studies in the field, we considered injury deaths of undetermined intent as suicides given that previous research has shown that these deaths are very likely to have resulted from suicide [33–35].

Prior to conducting the data linkage, we estimated the number of suicide deaths among CPP offspring between 1979 and 2016 that we would expect to observe based on historical suicide rates by age group and year obtained from the CDC mortality files [36]. We projected there would be between 285 and 304 deaths by suicide among CPP offspring (depending on the method of extrapolating to the oldest age group; see Supplemental Table 1 for details).

### Exposures

Collection of the exposure data was jointly conducted by trained nonphysician interviewers and physicians, beginning at the time of registration for prenatal care, at intervals of 4 weeks during the first 7 months of pregnancy, every 2 weeks at 8 months, and every week thereafter, using standardized protocols, forms, manuals, and codes. Throughout the initial and repeat prenatal visits, interviewers collected sociodemographic information, reproductive and gynecological history, recent and past medical history that needed treatment or hospitalizations, and family health and genetic history. Physicians reviewed the data collected by the interviewer, collected further details on past and recent medical history, completed initial prenatal examination and observations, and recorded the date, and listed any diagnoses unrelated to prenatal care that came to their attention. Medical and lay editing was subsequently carried out in conjunction with the participant’s complete hospital records by the obstetric coordinator or a board-certified obstetrician. Labor, birth, and neonatal information was based on direct observations and examinations of the baby made by either a nurse or a medical doctor (pediatrician, obstetrician).

#### Sociodemographic factors

Socioeconomic and demographic information was collected from the social history interview administered to mothers upon enrollment into the study during pregnancy. The following factors were included: race (White, Black or Other), family structure (a combination of marital status and whether father lives at home or is absent: both parents at home, single mother or married and father absent, or separated/widowed/divorced mother and father absent), household density (calculated as the number of people in the household at birth divided by the number of bedrooms in the household: <1 person, 1 to <1.5 persons, or ≥1.5 persons per bedroom), income relative to the US poverty threshold (categorized with percentiles 90th, 75th, 50th, 25th, 10th, and <10th), the highest level of parental education (high-school graduate or less [≤12 years], more than high school [>12 years]), parental occupation (maximum level in the household: non-manual, manual, or unemployed/student), and birth order (1st or only child, 2nd, 3rd, 4th or higher). The sex of the child was also included.

#### Pregnancy-related factors

The CPP collected detailed information on maternal health during pregnancy. Rather than examining every factor indiscriminately, and given the low prevalence of some conditions, we constructed broad-based measures of pregnancy complications, labor and delivery complications, and neonatal complications by adapting the Perinatal Risk Index developed by Marceau et al. [37] which has been used before to predict behavioral problems in childhood [38, 39]. Complication or risk scores have been used in previous studies to examine the risk of suicide death in offspring [40–42].

A full list of risk factors and associated scores for each of them is provided in Supplemental Table S2. Following Marceau et al. [37, 39] the pregnancy complication score included risk factors such as maternal age, multiple gestation, intrauterine growth, and maternal infections during pregnancy. The labor and delivery complication score included factors such as labor induction, labor length, cesarean delivery, and umbilical cord complications. Finally, the neonatal complication score included factors like gestational age, low birth weight, low Apgar scores, and neonatal infections.

Using Marceau et al.’s scheme, each risk factor was assigned a weight based on the harm it could cause to the fetus or neonate. Scores for individual factors (Supplemental Table S2) ranged from 3 (“Potentially but not clearly harmful or relevant”) to 6 (“Very great harm to or deviation in offspring”) [37, 39]. Conditions with scores 1 or 2 were considered not or not likely harmful or relevant and were given a score of 0. Complication scores were created by summing the weighted items in each domain based on these levels of risk and the number of risk factors endorsed and then categorized for analysis. For example, the pregnancy complication score had the following five categories: category 1 (scores 0–2), category 2 (scores 3–4), category 3 (scores 5–7), category 4 (scores 8–10), and category 5 (scores > 10). Consequently, someone with any harmful risk factor would be at least in category 2.

In addition to pregnancy-related complication scores, we also examined individual risk factors that have been associated with suicide behaviors in offspring in previous studies [15], most of which were included in the computation of complication scores. These included maternal age, maternal psychiatric history, birth weight, small for gestational age, and smoking during pregnancy.

### Statistical analyses

Using Stata SE 16 [43], we estimated the incidence rate per 100,000 person-years for the whole sample and within each category of each sociodemographic and obstetric variable examined. Cox proportional hazard models were used to investigate associations of sociodemographic, pregnancy-related factors, and the individual risk factors with suicide deaths occurring through 2016. Follow-up of participants started at each person’s age on January 1, 1979, when the National Death Index was established. Observations were censored: (a) on the date of death from causes other than suicide; or (b) at the end of 2016 if participants were still alive, thereby estimating cause-specific hazard ratios for suicide [44].

We set out to examine the contribution of risk factors within two domains to the risk of suicide mortality: sociodemographic factors and pregnancy-related factors, including both computed complication scores, and individual risk factors. After estimating unadjusted hazard ratios (HRs) for all risk factors, we estimated adjusted HRs for risk factors within each domain, and then carried over the significant risk factors within-domains to a fully adjusted model. Since most of the individual risk factors were included in the computation of complication scores, they were not included in the models with the complication scores. In sensitivity analyses, we repeated the sequence of models limited to deaths of intentional self-harm (i.e., excluding deaths by injury of undetermined intent). Finally, as an exploratory step, we conducted additional analyses among males and females separately, given sex differences in suicide risk, and tested time-varying effects of exposures on suicides occurring through age 30 and after age 30.

## Results

### Suicide incidence

Of the 49,853 participants submitted to the NDI for linkage, 3,555 matched to a death certificate with a probability score above the NDI cutoff to qualify as a true match. Of these, 288 (214 males, 74 females) were suicide deaths (incidence rate (IR) of 15.7 per 100,000 person-years, 95% CI 13.9–17.6), which included 175 (60.8%) deaths of intentional self-harm and 113 (39.2%) deaths of injury of undetermined intent. This number was close to our projection through 2016.

**Fig. 1:**
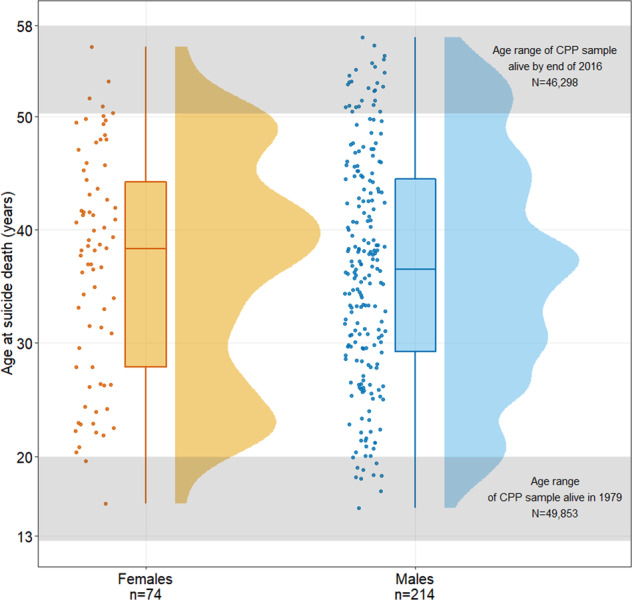
Distribution of age at the time of suicide death by sex. The lower shaded area represents the age range of the study sample at origin of follow-up; the upper shaded area represents the age range of the sample that was alive at the end of follow-up.

The median age of suicide death was 36 years for males (IQR = 29–44 years) and 38 years for females (IQR = 27–44), with few suicides occurring before age 20 (Fig. 1). Suicide deaths before age 19 were extremely rare, with only 12 cases (4.75% of total), and most of these occurring at 18 and 19 years of age.

Table 1 shows the frequency distribution of method of suicide for suicide deaths. The most common method of suicide was poisoning by solid or liquid substances (45%), followed by firearms and explosives (23%), and hanging, strangulation, and suffocation (19%). When examining deaths by intentional self-harm and by injury of undetermined intent separately, death by firearm was the most common method of deaths by intentional self-harm, accounting for 36% of cases, while poisoning accounted for 85% of deaths by injury of undetermined intent.

**Table 1:** Methods of suicide among suicide deaths in the Collaborative Perinatal Project cohort (*n* = 288).

Method	ICD codes		Men	Women	Total
	ICD-9	ICD-10	*n* (%)	*n* (%)	*n* (%)
Poisoning by solid or liquid substances^a^	E950; E980	X60–X65, X68–X69, Y10–Y15, Y18–Y19	85 (39.7)	44 (59.5)	129 (44.8)
Poisoning by gases in domestic use, other gases, and vapors	E951–E952, E981–E982	X66–X67, Y16–Y17	9 (4.2)	1 (1.4)	10 (3.5)
Hanging, strangulation, and suffocation	E953, E983	X70, Y20	45 (21)	10 (13.5)	55 (19.1)
Submersion (drowning)	E954, E984	X71, Y21	7 (3.3)	1 (1.4)	8 (2.8)
Firearms and explosives	E955, E985	X72–X75, Y22–Y25	52 (24.3)	13 (17.6)	65 (22.6)
Cutting and piercing instruments	E956; E986	X78, Y28	4 (1.9)	0 (0)	4 (1.4)
Jumping from high place	E957, E987	X80, Y30	5 (2.3)	1 (1.4)	6 (2.1)
Other and unspecified means	E958–E959, E988–E989	X76–X77, X79, X81–X84, Y26–Y27, Y29, Y31–Y34, Y87.0, Y87.2	7 (3.3)	4 (5.4)	11 (3.8)
Total			214 (74.3)	74 (25.7)	288 (100)

^a^Includes 1 case of suicide as contributing cause and 4 cases of undetermined injury death as contributing cause. For the remaining cases and methods, either suicide or undetermined injury death were the underlying cause of death.

Table 2 shows the distribution of sociodemographic and pregnancy-related factors examined in this study for suicide decedents and all other participants, with corresponding incidence rates of suicide per 100,000 person-years. Differences in incidence rates were especially evident for race and sex, in which white race and men had higher incidence rates of suicide. These were also higher for individuals with single-parent families, low parental education, manual occupations, higher birth order, pregnancy complications, maternal psychiatric history, and maternal smoking during pregnancy.

**Table 2:** Descriptive characteristics for sociodemographic and pregnancy-related factors of the suicide cases and non-cases.

	Non-cases, *n* (%)	Suicides, *n* (%)	Suicide incidence rate per 100,000 person-years (95%CI)
Race			
White	22551 (45.7)	171 (59.8)	20.3 (17.5, 23.6)
Black	23112 (46.8)	106 (37.1)	12.4 (10.3, 15.0)
Other	3702 (7.5)	9 (3.1)	6.5 (3.4, 12.6)
Sex of the child			
Male	24972 (50.4)	214 (74.3)	23.2 (20.3, 26.6)
Female	24542 (49.6)	74 (25.7)	8.1 (6.4, 10.1)
Family structure			
Both parents at home	37474 (77)	224 (78.1)	16.1 (14.1, 18.3)
Single or Married & Father absent	7125 (14.6)	32 (11.1)	12.1 (8.6, 17.1)
Sep/Widowed/Divorced & Father absent	4086 (8.4)	31 (10.8)	20.6 (14.5, 29.2)
Household density			
< 1 persons per bedroom	23249 (48)	128 (44.8)	14.8 (12.4, 17.6)
1–<1.5 persons per bedroom	8005 (16.5)	60 (21.0)	20.2 (15.7, 26.0)
≥1.5 persons per bedroom	17163 (35.5)	98 (34.3)	15.5 (12.7, 18.9)
Poverty ratio category			
90th percentile	4815 (10.5)	24 (8.8)	13.3 (8.9, 19.8)
75th percentile	7049 (15.4)	50 (18.3)	19.0 (14.4, 25.0)
50th percentile	11701 (25.6)	64 (23.4)	14.7 (11.5, 18.8)
25th percentile	10167 (22.2)	65 (23.8)	17.3 (13.6, 22.1)
10th percentile	6301 (13.8)	45 (16.5)	19.3 (14.4, 25.9)
<10th percentile	5687 (12.4)	25 (9.2)	11.9 (8.1, 17.6)
Parental education ≤ 12 years			
No	6622 (13.6)	19 (6.6)	7.7 (4.9, 12.0)
Yes	41982 (86.4)	268 (93.4)	17.2 (15.3, 19.4)
Parental occupation at birth			
Non-manual	17768 (36.5)	91 (31.7)	13.7 (11.2, 16.9)
None/student	3042 (6.3)	16 (5.6)	14.2 (8.7, 23.2)
Manual	27849 (57.2)	180 (62.7)	17.5 (15.1, 20.2)
Birth order			
1st	13382 (27.9)	58 (20.6)	11.6 (9.0, 15.0)
2nd	10301 (21.5)	57 (20.2)	14.8 (11.4, 19.2)
3rd	7567 (15.8)	51 (18.1)	18.1 (13.7, 23.8)
≥4th	16752 (34.9)	116 (41.1)	18.7 (15.6, 22.4)
Pregnancy complication score^a^			
0	2673 (5.4)	8 (2.8)	8.1 (4.0, 16.2)
3–4	11649 (23.5)	58 (20.1)	13.4 (10.4, 17.3)
5–7	13837 (27.9)	82 (28.5)	16.0 (12.9, 19.8)
8–10	9368 (18.9)	65 (22.6)	18.7 (14.6, 23.8)
>10	12038 (24.3)	75 (26)	16.8 (13.4, 21.1)
Labor and delivery complication score^b^			
0	13894 (28)	74 (25.7)	14.4 (11.5, 18.1)
3–4	15982 (32.2)	101 (35.1)	17.0 (14.0, 20.7)
5–7	7093 (14.3)	46 (16)	17.5 (13.1, 23.4)
>7	12596 (25.4)	67 (23.3)	14.3 (11.3, 18.2)
Neonatal complication score^c^			
0	11937 (24.1)	77 (26.7)	17.4 (13.9, 21.7)
3–4	16196 (32.7)	90 (31.3)	15.0 (12.2, 18.4)
5–8	11047 (22.3)	60 (20.8)	14.6 (11.4, 18.8)
>8	10385 (21.0)	61 (21.2)	15.9 (12.4, 20.4)
Mother age, years			
<20	11627 (23.5)	68 (23.6)	15.8 (12.4, 20.0)
20–24	18079 (36.5)	96 (33.3)	14.3 (11.7, 17.5)
25–29	10521 (21.2)	63 (21.9)	16.1 (12.6, 20.6)
30–34	5665 (11.4)	35 (12.2)	16.7 (12.0, 23.2)
≥35	3673 (7.4)	26 (9.0)	19.2 (13.0, 28.1)
Maternal psychiatric history			
No	45479 (91.8)	255 (88.5)	15.1 (13.4, 17.1)
Yes	4071 (8.2)	33 (11.5)	21.7 (15.4, 30.5)
Birth weight			
<1,500 g	269 (0.5)	2 (0.7)	20.0 (5.0, 80.0)
1,500–2,499 g	4816 (9.7)	29 (10.1)	16.3 (11.3, 23.5)
2,500–4,000 g	41868 (84.6)	236 (81.9)	15.2 (13.4, 17.2)
>4,000 g	2520 (5.1)	21 (7.3)	22.5 (14.7, 34.5)
Small for gestational age			
No	43342 (89.2)	250 (88.3)	15.5 (13.7, 17.6)
Yes	5244 (10.8)	33 (11.7)	16.9 (12.0, 23.8)
Smoking during pregnancy			
No	19180 (39.2)	86 (30.1)	12.1 (9.8, 14.9)
Yes	29815 (60.9)	200 (69.9)	18.1 (15.7, 20.8)

*CI* confidence interval

^a^Pregnancy complications include maternal age-related risk (below age <17 or >40), multiple gestation, intrauterine growth restriction, intrauterine growth acceleration, fetal heart rate deviations (e.g., abnormal heart rate/rythm), bleeding during pregnancy (e.g., placental abruption, early late pregnancy bleeding, and antepartum hemorrhage), placental abnormalities (e.g., single umbilical artery and placental previa), other fetal or placental conditions (e.g., premature rupture of membranes, oligohydramnios, and polyhydramnios), maternal conditions (e.g., preeclampsia, hypertension, albuminuria, and excessive weight gain), weight loss, hyperemesis, maternal hormonal and metabolic disorders (e.g., diabetes and hyper/hypothyroidism), appendicitis, neurological conditions (e.g., convulsive disorder), maternal infections (e.g., gonorrhea, syphilis, other STD, urinary infection, and pyelonephritis), amniocentesis, and maternal substance use (e.g., smoking during pregnancy, and drug habituation/addiction).

^b^Labor and delivery complications include induced labor, labor length (prolonged labor, precipitous delivery, and tocolysis), abnormal fetal presentation, cephalopelvic disproportion (eg., fetopelvic disproportion and placenta previa without bleeding), operative vaginal delivery (e.g., forceps or vacuum assisted vaginal delivery), cesarean delivery, fetal distress, meconium in amniotic fluid, umbilical cord complications during delivery (e.g., cord prolapse, nuchal, or body cord), placental abruption, and postpartum hemorrhage.

^c^Neonatal complications include deviations in gestational age (prematurity or post-term), low birth weight, circulatory system malformations (e.g., heart murmur), genital tract malformations (e.g., hydronephrosis), musculoskeletal malformations (e.g., craniosynostosis), congenital infections, hyperbilirubinemia, cephalohematoma, early neonatal complications (e.g., low Apgar scores, cyanosis, required resuscitation after birth, apnea, respiratory distress, required oxygen support), meconium aspiration syndrome, bradycardia, endocrinological disorders (e.g., hypocalcemia and hypoglycemia), neonatal infections (e.g., sepsis, pneumonia, and eye infection), hypothermia, and conditions requiring blood transfusion.

### Survival analysis of suicide mortality

The first column in Table 3 provides unadjusted hazard ratios for all hypothesized risk factors. When adjusting models within factor domains (i.e., sociodemographic factors, pregnancy-related complications scores, and selected individual risk factors; second to fourth columns in Table 3), estimates were similar to those in the unadjusted models. For sociodemographic factors, the strongest associations were found for race and sex of the child, with offspring of White race and men having a higher risk. Specifically, offspring of Black or other races had 0.51 (95% CI = 0.38, 0.67) and 0.27 (95%CI = 0.14, 0.54) times hazard of mortality by suicide, respectively, than offspring of White race; and the hazard in women was 0.34-fold (95%CI = 0.25, 0.44) compared to men. Offspring of parents with no more than high-school education (HR = 2.16; 95%CI = 1.32, 3.51) and with manual occupations (HR = 1.46; 95%CI = 1.09, 1.95) were also at higher risk of suicide. Children who were 4th born or later had 1.53 times (95%CI = 1.04, 2.26) higher hazard of suicide than 1st born and only-child offspring.

**Table 3:** Results of survival analyses of the associations of sociodemographic factors, pregnancy, birth and neonatal Complication scores, and other individual risk factors with suicide mortality.

	Unadjusted HR (95%CI)	Adjusted for sociodemographic risk factors HR (95%CI)^a^	Adjusted for pregnancy-related complication scores HR (95%CI)^b^	Adjusted for pregnancy-related individual risk factors HR (95%CI)^c^	Adjusted for sociodemographic risk factors and pregnancy-related complication scores HR (95%CI)^d^	Adjusted for sociodemographic risk factors and pregnancy-related individual risk factors HR (95%CI)^e^
Sociodemographic factors						
*Race*						
White	Ref.	Ref.			Ref.	Ref.
Black	0.61 (0.48, 0.78)	0.51 (0.38, 0.67)			0.49 (0.38, 0.64)	0.50 (0.38, 0.66)
Other	0.32 (0.16, 0.63)	0.27 (0.14, 0.54)			0.28 (0.14, 0.54)	0.29 (0.15, 0.57)
Sex of the child						
Male	Ref.	Ref.			Ref.	Ref.
Female	0.35 (0.27, 0.45)	0.34 (0.25, 0.44)			0.34 (0.26, 0.44)	0.34 (0.26, 0.44)
*Family structure*						
Both parents at home	Ref.	Ref.				
Single or married & father absent	0.75 (0.52, 1.09)	1.04 (0.65, 1.67)				
Sep/widowed/divorced & father absent	1.28 (0.88, 1.86)	1.22 (0.81, 1.83)				
*Household density*						
<1 persons per bedroom	Ref.	Ref.				
1–<1.5 persons per bedroom	1.37 (1.01, 1.86)	1.24 (0.88, 1.74)				
≥1.5 persons per bedroom	1.05 (0.81, 1.37)	1.01 (0.74, 1.38)				
*Poverty ratio category*						
90th percentile	Ref.	Ref.				
75th percentile	1.43 (0.88, 2.33)	1.19 (0.72, 1.96)				
50th percentile	1.11 (0.69, 1.77)	0.83 (0.50, 1.37)				
25th percentile	1.31 (0.82, 2.09)	0.96 (0.56, 1.63)				
10th percentile	1.46 (0.89, 2.40)	1.05 (0.59, 1.87)				
<10th percentile	0.90 (0.51, 1.58)	0.60 (0.31, 1.16)				
*Parental education* *≤* *12 years*						
No	Ref.	Ref.			Ref.	Ref.
Yes	2.25 (1.41, 3.59)	2.16 (1.32, 3.51)			2.23 (1.38, 3.62)	2.19 (1.35, 3.55)
*Parental occupation at birth*						
Non-manual	Ref.	Ref.			Ref.	Ref.
None/student	1.04 (0.61, 1.77)	1.45 (0.72, 2.93)			1.52 (0.85, 2.72)	1.58 (0.87, 2.83)
Manual	1.27 (0.99, 1.64)	1.46 (1.09, 1.95)			1.38 (1.05, 1.82)	1.40 (1.06, 1.85)
*Birth order*						
1st	Ref.	Ref.			Ref.	Ref.
2nd	1.28 (0.89, 1.84)	1.29 (0.87, 1.91)			1.29 (0.89, 1.87)	1.27 (0.88, 1.84)
3rd	1.56 (1.07, 2.27)	1.36 (0.89, 2.09)			1.50 (1.02, 2.21)	1.48 (1.01, 2.18)
≥4th	1.61 (1.18, 2.21)	1.53 (1.04, 2.26)			1.52 (1.10, 2.11)	1.49 (1.07, 2.06)
Complication scores						
*Pregnancy complication score*						
0	Ref.		Ref.		Ref.	
3–4	1.66 (0.79, 3.48)		1.65 (0.79, 3.46)		1.84 (0.84, 4.04)	
5–7	1.97 (0.96, 4.08)		1.97 (0.95, 4.07)		2.06 (0.95, 4.45)	
8–10	2.31 (1.11, 4.82)		2.32 (1.11, 4.83)		2.36 (1.08, 5.16)	
>10	2.08 (1.00, 4.31)		2.09 (1.01, 4.35)		2.16 (0.99, 4.69)	
*Labor / Delivery complication score*						
0	Ref.		Ref.			
3–4	1.18 (0.87, 1.59)		1.16 (0.86, 1.57)			
5–7	1.22 (0.84, 1.76)		1.19 (0.82, 1.72)			
>7	0.99 (0.71, 1.38)		0.96 (0.69, 1.33)			
*Neonatal complication score*						
0	Ref.		Ref.			
3–4	0.86 (0.63, 1.17)		0.86 (0.63, 1.17)			
5–8	0.84 (0.60, 1.18)		0.83 (0.59, 1.17)			
>8	0.91 (0.65, 1.28)		0.89 (0.64, 1.25)			
Individual risk factors (included in complication scores)						
*Mother age, years*						
<20	1.10 (0.81, 1.51)			1.16 (0.85, 1.59)		
20–24	Ref.			Ref.		
25–29	1.13 (0.82, 1.55)			1.10 (0.80, 1.53)		
30–34	1.17 (0.79, 1.72)			1.11 (0.75, 1.65)		
≥35	1.34 (0.87, 2.07)			1.31 (0.84, 2.03)		
*Maternal psychiatric history*						
No	Ref.			Ref.		
Yes	1.44 (1.00, 2.06)			1.40 (0.97, 2.02)		
*Birth weight*						
<1,500 g	1.32 (0.33, 5.31)			-		
1,500–2,499 g	1.07 (0.73, 1.58)			0.96 (0.61, 1.53)		
2,500–4,000 g	Ref.			Ref.		
>4,000 g	1.48 (0.95, 2.32)			1.45 (0.91, 2.29)		
*Small for gestational age*						
No	Ref.			Ref.		
Yes	1.09 (0.76, 1.57)			1.13 (0.73, 1.73)		
*Smoking during pregnancy*						
No	Ref.			Ref.		Ref.
Yes	1.50 (1.16, 1.93)			1.50 (1.16, 1.94)		1.28 (0.99, 1.66)

*HR* hazard ratio, *CI* confidence interval, *Ref*. reference

^a^*N* = 44,941, including 268 suicide cases.

^b^*N* = 49,714, including 288 suicide cases.

^c^*N* = 48,193, including 281 suicide cases.

^d^*N* = 47,731, including 281 suicide cases.

^e^*N* = 47,516, including 278 suicide cases.

For pregnancy-related factors, higher pregnancy complication score, but not labor or neonatal complication scores, was associated with a higher risk of suicide in the offspring after adjusting for each other. Offspring of pregnancies in the top 2 categories of the pregnancy complications score had a twofold increase in the risk of suicide (HR = 2.32; 95%CI = 1.11, 4.83 and HR = 2.09; 95%CI = 1.01, 4.35) compared to offspring of pregnancies in the lowest category of the pregnancy complications score. In addition, among pregnancy-related individual risk factors, smoking during pregnancy (HR = 1.50; 95%CI = 1.16, 1.94) was associated with a higher risk of offspring suicide after adjusting for the other individual risk factors.

In the final adjusted models, the magnitude of the associations was similar to the estimates in the domain-specific models. Notably, offspring of pregnancies with high complication scores had twofold increases in suicide risk (HR = 2.36; 95%CI = 1.08, 5.16 and HR = 2.16; CI = 0.99, 4.69 in the top two categories); independent of that increased risk, offspring of parents with no more than 12 years of education (HR = 2.23; 95%CI = 1.38, 3.62) and with manual occupations (HR = 1.38; 95%CI = 1.05, 1 .82) also had an elevated risk of suicide mortality. Suicide risk also varied by race (lower risk for non-White vs. White), sex (lower risk for females), and birth order (higher risk for later-born offspring). The estimate for smoking during pregnancy decreased after adjusting for sociodemographic factors, though suggested an effect in the same direction (HR = 1.28; 95%CI = 0.99, 1.66)

### Sensitivity analysis

The results with only deaths by intentional self-harm (*N* = 175) counted as suicides were similar to those found using all cases (i.e., including deaths by injury of undetermined intent), with the exception of birth order, which was no longer associated with suicide (Table S3). Specifically, in the fully adjusted models, a higher risk of suicide was found for offspring of White race (Black vs White race, HR = 0.41; 95%CI = 0.29, 0.58, and Other vs White race, HR = 0.24; 95%CI = 0.1, 0.59), males (Females vs Males, HR = 0.34; 95%CI = 0.24, 0.49), lower parental education (HR = 1.82; 95%CI = 1.05, 3.15), manual occupation (HR = 1.49; 95%CI = 1.05, 2.10), pregnancy complication top scores (HR = 6.41; 95%CI = 1.56, 26.39, and HR = 4.09; 95%CI = 0.99, 16.94), and smoking during pregnancy (HR = 1.40; 95%CI = 0.99, 1 .96).

### Sex differences and time-varying effects

The results examining risk factors by sex are exploratory and should be interpreted with caution. There was no evidence of sex interactions with any of the risk factors (Table S4). For males, higher risk of suicide was associated with more risk factors than for females. However, the effect sizes of those factors and its direction in females suggested similar associations than those seen males, especially for low parental education (HR = 2.68; 95%CI = 0.98, 7.34), and smoking during pregnancy (HR = 1.34; 95%CI = 0.82, 2.17). Parental occupation was not associated with suicide risk when examined separately by sex; however, effect sizes and its direction were comparable to those find in the whole sample (Males, HR = 1.26; 95%CI = 0.94, 1.68; Females, HR = 1.35; 95%CI = 0.82, 2.25).

Analysis of time-varying effects (Table S5) with suicides occurring through age 30 (*n* = 82) and after age 30 (*n* = 206) suggested that having a separated/divorced/widowed mother increases the risk of suicide mortality through age 30 (HR = 2.63; 95%CI = 1.53, 4.51) but not at older ages (HR = 0.79; 95%CI = 0.46, 1.36) whereas the opposite pattern was seen for maternal psychiatric history (before/at age 30: HR = 1.15; 95%CI = 0.55, 2.39; after age 30: HR = 1.57; 95%CI = 1.03, 2.39).

## Discussion

Using a large pregnancy cohort from the United States, we examined whether sociodemographic and pregnancy-related factors were associated with the risk of suicide mortality in offspring followed through middle adulthood. We found that sociodemographic factors including male sex, White race, low parental education, manual parental occupation, and later birth order were associated with higher suicide risk. Higher rates of pregnancy complications and smoking during pregnancy were independently associated with suicide risk. In contrast, birth and neonatal complications were not associated with the risk of suicide. Similar results were obtained when excluding deaths by undetermined intent from the analyses.

Consistent with evidence supporting the developmental origins of mental disorders [13, 45] and the results from a recent meta-analysis [15], our findings highlight that vulnerability to suicide is established early in development. Pregnancy complications, but not delivery or neonatal complications, were associated with increased risk of suicide in offspring through middle adulthood, mirroring previous findings on suicide attempts in young people [25]. Maternal smoking during pregnancy has also been associated to an increased risk of suicide death in offspring in a large study [46], as has birth order, with later-born siblings having a higher risk for suicide [19, 47–54].

Low parental education is one of the sociodemographic factors that has been more consistently associated with suicide mortality in offspring [19, 46, 47]. We also found an association between parental occupation at birth and suicide in offspring. Whereas a study using a Scottish birth cohort also found an increased risk of suicide mortality in the offspring of parents with skilled and unskilled occupations compared to those with professional occupations [51], other studies that examined parental occupation at birth as a risk factor have not found this association [18, 48, 55–57]. The reason for this inconsistency is unclear; it is possible that the latter studies were not powered to detect an association between parental occupation at birth and suicide death in offspring, since the number of suicide cases in these studies ranged from 7 to 161, whereas the study that found an association had 1,464 suicides cases [51]. A further possibility is that our study was conducted in the US, which lacks universal healthcare coverage, in contrast to European countries. Thus, in the US, lower parental occupation is linked with a broad range of social determinants of health including healthcare during pregnancy, lack of paid parental leave [58], and access to medical resources during early childhood [59].

We found that sociodemographic and pregnancy risk factors are independently associated with suicide mortality. There are likely two categories of mechanisms underlying this vulnerability: neurodevelopmental effects and intergenerational transmission of social stressors. Studies in animals and humans suggest that maternal stress and fetal undernutrition might impact brain development and HPA-axis reactivity, both in-utero, and in childhood and adolescence [13, 60]. These pathophysiological changes, along with genetic, epigenetic, and gene-environment interaction effects, might increase the odds of psychopathology, cognitive deficits, and impulsive behavior. Together, the presence of these factors might increase vulnerability to suicide under certain circumstances [5, 6, 61]. Indeed, there is some evidence to suggest that the association between prenatal circumstances and suicide mortality might be partially mediated by the presence of affective disorders in adulthood [62]. In addition, some of the sociodemographic factors examined in the current study are likely to continue into childhood (e.g., low parental education, parental occupation) and therefore impact early childhood environment directly as well as adulthood socioeconomic status [63].

An important question is whether the associations between early life exposures and suicide mortality are causal or not. It could be that these associations are explained by shared environmental and genetic family components. Our study included maternal psychiatric history as exposure, which was associated with suicide risk in the unadjusted model. However, the strength of the association faded when adjusting for sociodemographic factors, suggesting that the associations found with other factors were not explained by maternal psychiatric history. Nevertheless, we lacked information on suicidal behaviors and paternal psychiatric history. Family aggregation of suicidal behaviors exists, and family history of self-harm is associated with suicide behaviors in offspring [64], with both environmental and genetic components contributing to intergenerational transmission [65]. Previous studies have tried to address causality using sibling comparison designs. However, the results of these studies have been inconsistent depending on the domain of risk factor examined. For example, after accounting for shared family factors, some studies have reported attenuated but significant associations between sociodemographic factors and suicide involving maternal age and birth order [47, 52]. In other studies, associations between fetal growth and suicide risk disappeared after accounting for shared family factors [66, 67], though a recent study found evidence for causal effect of birth weight on suicide attempt using a two-sample Mendelian randomization approach [68].

The results of this study need to be interpreted in light of its limitations. First, since the US National Death Index was established in 1979, any suicide deaths occurring between the end of the CPP data collection (when children were 7–8 years of age) and 1979 were not observed. The age range of CPP participants at the start of follow-up in 1979 was from nearly 13 to nearly 20 years old, with more than 50% of participants being under 15. Although this is an earlier age than most studies examining early life risk factors of suicide death [15], we would have expected a very small number of suicide deaths occurring during the unobserved person-years, thus unlikely to change the results. Second, although the maximum age of CPP participants that were alive by the end of the follow-up period was 57 years, with virtually all participants followed through age 50, the current study does not address potential early risk factors of suicide in older adulthood, when suicide rates increase [3]. Therefore, it is possible that results could differ if the study were extended into older adulthood. Second, while we had information on maternal psychiatric history, the CPP did not collect data on paternal psychiatric history and parental history of self-harm, which could be potential confounders and have been shown to be associated with offspring risk of suicidal behaviors [65]. Future studies would benefit from including these factors to determine how much the risk of suicide death among offspring can be attributed to parental mental health and history of suicidal behaviors and account for this risk while examining sociodemographic and pregnancy-related factors. Third, as in all studies of suicide mortality that identify suicide deaths using death certificates, there is potential misclassification due to variations in assigning cause of death across jurisdictions, especially in deaths caused by drug intoxication [69]. In the current study, this is evidenced by the high rates of deaths by poisoning labeled as death of undetermined intent (85%) compared to those labeled as death of intentional self-harm (19%). Most studies on suicide consider deaths of undetermined intent as suicides since these deaths are very likely to have resulted from suicide [35]. Our sensitivity analysis counting only deaths by intentional self-harm as suicides yielded similar results. A fourth limitation is that we identified suicide deaths through a probabilistic linkage to the US National Death Index, meaning there was likely some misclassification of vital status. However, probability score matching in the NDI has been used before with high sensitivity (>85%) and specificity (>90%) [70], even with the absence of social security numbers (SSN) [30, 32], making the NDI the most reliable source of mortality data in the US [70].

Our findings support the developmental origin of suicide mortality. Both sociodemographic and pregnancy-related risk factors are independently associated with the risk of suicide death in the offspring. As it has been done with etiological models of several psychiatric conditions, etiological models of suicide should be expanded to include developmental processes starting as early as prenatal life. Whether or not the pathways implicated by our results are specific to suicide or are shared with the developmental pathways underlying multiple forms of psychopathology is unclear; however, findings that more than half of suicides occur outside of the context of major mental disorders would suggest that some suicide-specific vulnerability exists [71].

Above we argued that advancing knowledge of suicide etiology to inform prevention requires a greater understanding of distal processes. Invariably, proximal risk factors will be more predictive of imminent risk than distal risk factors that may have occurred decades earlier, and so prevention of acute suicide risk likely necessitates an emphasis on proximal risk factors. However, a developmental approach to suicide etiology and prevention that also incorporates distal factors could expand opportunities to prevent suicide once the mechanisms underlying distal factors are understood. In theory, the presence of proximal factors, such as having depression, in the context of vulnerability established years earlier—such as exposure to pregnancy complications as shown here—might be more strongly associated with suicide than depression in the absence of this prior vulnerability, and if so, that knowledge could be used to develop a more precise risk assessment among individuals with psychiatric conditions [72]. Future research is needed to examine the mechanisms underlying the association between early risk factors and suicide mortality later in life that incorporates information about factors more proximal to time of death that might inform us on causal pathways [15].

The findings of the current and previous studies underscore the health benefits of prenatal care, including physical and mental health, and of policies to mitigate the socioeconomic burdens of mothers and families [73]. Early child intervention programs have long-term benefits for reducing many of the environmental, behavioral, and academic risk factors of suicide [74, 75], and even suicidal behaviors in young adulthood [76]. Extending early interventions even earlier in development could be beneficial for a broad range of outcomes including behavioral problems [11, 12], depression [8, 9], and ultimately suicide.

## Availability of data

Data from the Collaborative Perinatal Project is publicly available at https://www.archives.gov/research/electronic-records/nih.html (National Archives Identifier: 606622). Data from the National Death Index cannot be shared.

## Supplementary information

Supplemental material
