# Supplementary material for: Prenatal origins of suicide mortality: A prospective cohort study in the United States
Source: Transl Psychiatry. 2022 Jan 10;12:14. doi: 10.1038/s41398-021-01777-x (PMC8748551; doi:10.1038/s41398-021-01777-x)
Supplement: Supplementary file 1 — Supplemental material [file 41398_2021_1777_MOESM1_ESM.docx]

**Prenatal Origins of Suicide Mortality: A Prospective Cohort Study in the United States**

**Supplemental Material**


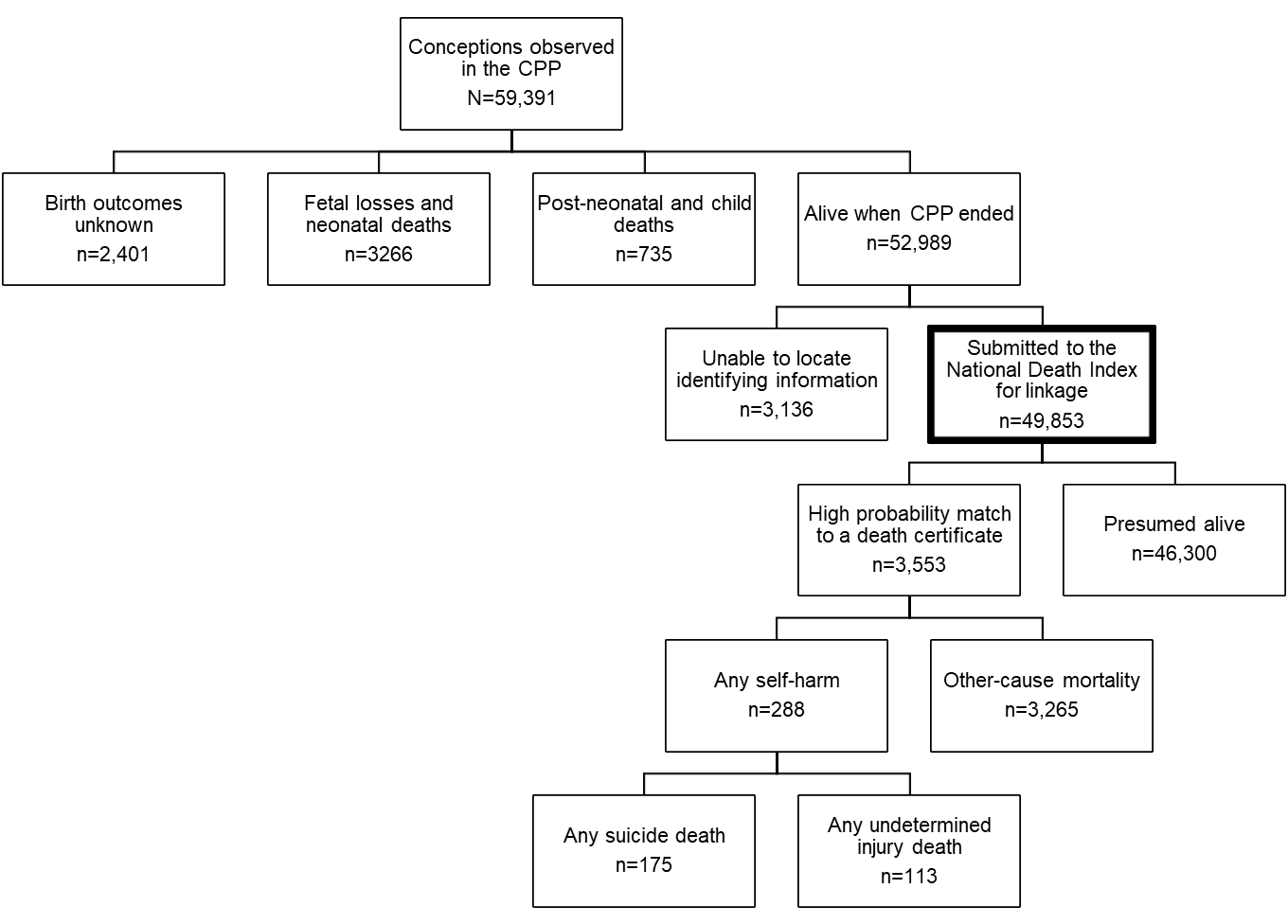


**Figure S1. Flowchart of Collaborative Perinatal Project offspring linkage study. Box with bold border indicates the sample included in the current study**

**Projected number of suicide deaths among CPP offspring**

Historical suicide rates by age group and year (in 3 broad categories so as to smooth over year-to-year variation but still account for secular trends) were obtained from the CDC mortality files (<http://wonder.cdc.gov/mortSQL.html>) to estimate the expected number of suicide deaths among CPP offspring. The results are shown in **Table S1.** We first calculated the number person-years that would be observed among the 52,966 surviving CPP offspring through 2016 – this was 2,012,708. The second column in Table S1 extrapolates the age distribution of CPP offspring through the calendar years 1968-2016. For example, between 1968-1978, 267,755 person-years were observed among children between the ages 5-9. The suicide rate per 100,000 during that time period was 0.011 (third column). Multiplying 267,755 by 0.011/100,000 gives 0.03, which we round to 0 expected deaths. We repeated the procedure for each age group in each of the 3 calendar periods. Of note, the older participants in the CPP were 57 years old by the end of 2016; specifically, 20% of the participants were between 55 and 57. However, the CDC mortality files only provide 10-year ranges at those ages. Therefore, we projected suicides including and then excluding the age range of 55-64 years. The total number of expected deaths including the age range of 55-64 was 304. The total number of expected deaths excluding the age range of 55-64 was 285. The difference in projection is 19 deaths. Although unlikely, assuming the same number of suicide deaths at each age between 55-64 in the population, and only taking 20%, these would be nearly 4 deaths for those aged 55-57, which added to the 285 would be 289 suicide deaths projected. A caveat to these projections is that they do not remove from the number of person-years at risk deaths that occurred from other causes; however, given the relatively young age of the cohort, we anticipate the overestimate to be rather small. For example, a projection of all-cause mortality in the 3 age groups represented between 1968-1978 yields 272 deaths. Removing the person-years following these 272 deaths from the denominator of person-years in that time span (582,626) would have a negligible effect on the projections.

**Table S1. Projected suicide deaths among CPP offspring through 2016**

| **Age group by calendar year** | **Number of person-years** | **Suicide rate/100,000 from CDC** | **Projected suicide deaths** |
| --- | --- | --- | --- |
| **1968-1978 ^a^** |  |  |  |
| 5-9 | 267,755 | 0.011 | 0.0 |
| 10-14 | 243,730 | 0.731 | 1.8 |
| 15-19 | 71,141 | 6.891 | 4.9 |
| **1979-1998** |  |  |  |
| 10-14 | 21,100 | 1.395 | 0.3 |
| 15-19 | 193,689 | 9.768 | 18.9 |
| 20-24 | 264,830 | 15.124 | 40.1 |
| 25-34 | 508,560 | 15.162 | 77.1 |
| 35-44 | 71,141 | 15.118 | 10.8 |
| **1999-2016** |  |  |  |
| 25-34 | 21,100 | 13.749 | 2.9 |
| 35-44 | 458,519 | 15.702 | 72.0 |
| 45-54 | 314,871 | 17.887 | 56.3 |
| 55-64 ^b^ | 158,898 | 16.036 | 25.5 |
| **Total** | **2,012,708** |  | **303.8 (including 55-64)** |
|  |  |  | **285.1 (excluding 55-64)** |
| ^a^ Projections for 1968-1978 are shown, but data for these years are not available in the US National Death Index. Therefore, the totals in the last rows cover 1979-2016 only.  ^b^ The oldest participants were 57 years old in 2016. However, CDC data only provides these age ranges. | | | |

**Table S2. Risk factors included in the risk scores for pregnancy, labor and delivery, and neonatal complications, along with the score assigned if the risk factor is present. The total score for each domain (i.e., pregnancy, labor and delivery, and neonatal) is the sum of assigned scores.**

| **Pregnancy**  **Complications** | | **Labor and delivery**  **complications** | | **Neonatal**  **complications** | |
| --- | --- | --- | --- | --- | --- |
| **Risk factor included** | **Score assigned if present** | **Risk factor included** | **Score assigned if present** | **Risk factor included** | **Score assigned if present** |
| Multiple pregnancy | 3 | Labor induction | 3 | Prematurity, postmaturity, low birthweight | 4 if postmaturity (gestational age >42 weeks) or low birthweight (<2500g)  5 if prematurity (gestational age <37 weeks) |
| Intrauterine growth restriction (<10^th^ percentile for gestational age) | 3 | Prolonged labor, precipitous delivery, and tocolysis | 3 if tocolysis  5 if prolonged labor or precipitous delivery | Circulatory system malformations | 3 if heart murmur |
| Intrauterine growth acceleration (>10^th^ percentile for gestational age) | 3 | Abnormal fetal presentation | 4 | Genital tract malformations | 3 if hydronephrosis |
| Fetal heart rate deviations (abnormal heart rate or abnormal heart rhythm) | 3 | Cephalopelvic distortion (fetopelvic disproportion, placenta previa without bleeding) | 4 | Musculoskeletal malformations | 4 if craniosynostosis |
| Bleeding during pregnancy | 4 if bleeding late in pregnancy or antepartum hemorrhage  5 if placental abruption or early bleeding | C-section, forceps,  vacuum delivery | 3 if vacuum-assisted delivery  4 if cesarean delivery or forceps-assisted delivery | Congenital infections | 5 |
| Placental conditions | 3 if placenta previa  4 if single umbilical artery | Fetal distress and meconium in amniotic fluid | 4 if meconium in amniotic fluid  5 if fetal distress | Hyperbilirubinemia | 4 |
| Other feto-placental conditions | 3 if polyhydramnios  4 if oligohydramnios  5 if premature rupture of membranes | Cord prolapse, cord around neck/body | 4 if cord around body, cord tight around neck, cord knotted around neck tight/loose  6 if cord prolapse | Cerebral hemorrhage | 4 |
| Maternal disorders | 3 if hypertension, albuminuria, or excessive weight gain  4 if preeclampsia | Placental abruption and postpartum hemorrhage | 4 if postpartum hemorrhage  5 if placental abruption | Early period general condition | 4 if cyanosis, required resuscitation after birth, apnea, respiratory distress, required oxygen support  5 if Apgar score at 1 or 5 min <7 |
| Low maternal weight gain, hyperemesis, poor nutrition | 4 if hyperemesis gradivarum or weight loss |  |  | Lung disorders | 5 if aspiration |
| Other maternal circulatory disorders | 3 if hypotension |  |  | Arrythmia | 5 if bradycardia |
| Noninfectious maternal respiratory disorders | 3 if bronchial asthma |  |  | Endocrinological disorders | 4 if hypocalcemia or hypoglycemia |
| Maternal hormonal and metabolic disorders | 3 if hypothyroidism  4 if diabetes, or hyperthyroidism |  |  | New neonatal infections | 5 if pneumonia or eye infection  6 if sepsis |
| Maternal gastrointestinal tract disorders | 4 if appendicitis |  |  | General disorders, signs and symptoms | 4 if low body temperature |
| Neurological conditions | 5 if convulsive disorder |  |  | Blood transfusion | 4 |
| Maternal infections | 3 if other sexually transmitted diseases, urinary infection, or pyelonephritis  4 if gonorrhea, or syphilis |  |  |  |  |
| Amniocentesis | 3 |  |  |  |  |
| Maternal age <17 years or >40 years | 4 |  |  |  |  |
| Maternal substance use | 3 if smoking during pregnancy  4 if drug habituation or addiction |  |  |  |  |
| Each risk factor had a score assigned based on the harm it could cause to the fetus ranging from 3 (“Potentially but not clearly harmful or relevant”) to 6 (“Very great harm to or deviation in offspring”). | | | | | |

**Table S3. Results of survival analyses of the Associations of Sociodemographic Factors, Pregnancy, Birth and Neonatal Complication Scores, and other Individual Risk Factors with Suicide Mortality (Intentional Self-Harm only)**

|  | **Unadjusted**  **HR (95%CI)** | **Adjusted for sociodemographic risk factors**  **HR (95%CI)^1^** | **Adjusted for pregnancy-related complication score**  **HR (95%CI)^2^** | **Adjusted for pregnancy-related individual risk factors**  **HR (95%CI)^3^** | **Adjusted for sociodemographic risk factors and pregnancy-related complication scores**  **HR (95%CI)^4^** | **Adjusted for sociodemographic risk factors and pregnancy-related individual risk factors**  **HR (95%CI)^5^** |
| --- | --- | --- | --- | --- | --- | --- |
| ***SOCIODEMOGRAPHIC FACTORS*** |  |  |  |  |  |  |
| ***Race*** |  |  |  |  |  |  |
| White | Ref. | Ref. |  |  | Ref. | Ref. |
| Black | 0.47 (0.34, 0.65) | 0.38 (0.26, 0.55) |  |  | 0.41 (0.29, 0.58) | 0.43 (0.30, 0.61) |
| Other | 0.27 (0.11, 0.65) | 0.23 (0.09, 0.56) |  |  | 0.24 (0.1, 0.59) | 0.25 (0.10, 0.63) |
| **Sex of the child** |  |  |  |  |  |  |
| Male | Ref. | Ref. |  |  | Ref. | Ref. |
| Female | 0.35 (0.25, 0.49) | 0.33 (0.23, 0.47) |  |  | 0.34 (0.24, 0.49) | 0.35 (0.25, 0.49) |
| **Family structure** |  |  |  |  |  |  |
| Both parents at home | Ref. | Ref. |  |  | Ref. | Ref. |
| Single or Married & Father absent | 0.67 (0.40, 1.11) | 1.16 (0.64, 2.11) |  |  | 0.82 (0.47, 1.45) | 0.83 (0.47, 1.47) |
| Sep/Widowed/Divorced & Father absent | 1.58 (1.02, 2.47) | 1.68 (1.04, 2.71) |  |  | 1.49 (0.94, 2.34) | 1.48 (0.94, 2.34) |
| **Household density** |  |  |  |  |  |  |
| < 1 persons per bedroom | Ref. |  |  |  |  |  |
| 1-<1.5 persons per bedroom | 1.12 (0.74, 1.69) | 1.14 (0.72, 1.8) |  |  |  |  |
| ≥1.5 persons per bedroom | 1.03 (0.74, 1.44) | 1.15 (0.78, 1.7) |  |  |  |  |
| **Poverty ratio category** |  |  |  |  |  |  |
| 90^th^ percentile | Ref. |  |  |  |  |  |
| 75^th^ percentile | 1.34 (0.76, 2.36) | 1.15 (0.64, 2.07) |  |  |  |  |
| 50^th^ percentile | 0.92 (0.53, 1.61) | 0.75 (0.41, 1.36) |  |  |  |  |
| 25^th^ percentile | 0.80 (0.45, 1.44) | 0.68 (0.35, 1.31) |  |  |  |  |
| 10^th^ percentile | 1.21 (0.67, 2.19) | 1 (0.5, 2) |  |  |  |  |
| <10^th^ percentile | 0.77 (0.39, 1.50) | 0.52 (0.23, 1.19) |  |  |  |  |
| **Parental education** ≤ **12 years** |  |  |  |  |  |  |
| No | Ref. | Ref. |  |  | Ref. | Ref. |
| Yes | 1.69 (1.00, 2.87) | 1.75 (1.00, 3.06) |  |  | 1.82 (1.05, 3.15) | 1.76 (1.02, 3.06) |
| **Parental occupation at birth** |  |  |  |  |  |  |
| Non-manual | Ref. | Ref. |  |  | Ref. | Ref. |
| None/student | 0.81 (0.39, 1.70) | 1.32 (0.52, 3.34) |  |  | 1.34 (0.59, 3.05) | 1.41 (0.62, 3.22) |
| Manual | 1.20 (0.87, 1.65) | 1.58 (1.09, 2.30) |  |  | 1.49 (1.05, 2.10) | 1.52 (1.07, 2.16) |
| **Birth order** |  |  |  |  |  |  |
| 1^st^ | Ref. | Ref. |  |  |  |  |
| 2^nd^ | 1.41 (0.90, 2.20) | 1.37 (0.86, 2.19) |  |  |  |  |
| 3^rd^ | 1.34 (0.82, 2.19) | 1.20 (0.70, 2.05) |  |  |  |  |
| ≥4^th^ | 1.40 (0.93, 2.09) | 1.31 (0.81, 2.14) |  |  |  |  |
| ***COMPLICATION SCORES*** |  |  |  |  |  |  |
| **Pregnancy complication score** |  |  |  |  |  |  |
| 0 | Ref. |  | Ref. |  | Ref. |  |
| 3-4 | 3.32 (0.79, 13.91) |  | 3.32 (0.79, 13.90) |  | 3.29 (0.79, 13.80) |  |
| 5-7 | 5.11 (1.24, 20.95) |  | 5.12 (1.25, 21.01) |  | 4.61 (1.12, 18.92) |  |
| 8-10 | 7.11 (1.73, 29.24) |  | 7.18 (1.75, 29.52) |  | 6.41 (1.56, 26.39) |  |
| >10 | 4.55 (1.10, 18.81) |  | 4.61 (1.11, 19.10) |  | 4.09 (0.99, 16.94) |  |
| **Labor / Delivery complication score** |  |  |  |  |  |  |
| 0 | Ref. |  | Ref. |  |  |  |
| 3-4 | 0.97 (0.66, 1.42) |  | 0.95 (0.65, 1.39) |  |  |  |
| 5-7 | 1.06 (0.66, 1.69) |  | 1.04 (0.65, 1.66) |  |  |  |
| >7 | 0.92 (0.61, 1.39) |  | 0.88 (0.58, 1.33) |  |  |  |
| **Neonatal complication score** |  |  |  |  |  |  |
| 0 | Ref. |  | Ref. |  |  |  |
| 3-4 | 0.92 (0.62, 1.36) |  | 0.92 (0.62, 1.36) |  |  |  |
| 5-8 | 0.86 (0.56, 1.34) |  | 0.85 (0.55, 1.32) |  |  |  |
| >8 | 0.97 (0.63, 1.50) |  | 0.95 (0.61, 1.47) |  |  |  |
| ***INDIVIDUAL RISK FACTORS***  ***(Included in complication scores)*** |  |  |  |  |  |  |
| **Mother age, years** |  |  |  |  |  |  |
| <20 | 1.28 (0.87, 1.89) |  |  | 1.35 (0.91, 2.00) |  |  |
| 20-24 | Ref. |  |  | Ref. |  |  |
| 25-29 | 1.10 (0.73, 1.68) |  |  | 1.08 (0.71, 1.65) |  |  |
| 30-34 | 1.20 (0.73, 1.98) |  |  | 1.14 (0.68, 1.90) |  |  |
| ≥35 | 1.41 (0.81, 2.47) |  |  | 1.41 (0.81, 2.46) |  |  |
| **Maternal psychiatric history** |  |  |  |  |  |  |
| No | Ref. |  |  | Ref. |  |  |
| Yes | 1.43 (0.90, 2.28) |  |  | 1.37 (0.86, 2.19) |  |  |
| **Birth weight** |  |  |  |  |  |  |
| <1,500g | - |  |  | - |  |  |
| 1,500-2,499g | 1.19 (0.73, 1.92) |  |  | 0.85 (0.47, 1.52) |  | 1.27 (0.77, 2.08) |
| 2,500-4,000g | Ref. |  |  | Ref. |  | Ref. |
| >4,000g | 1.91 (1.14, 3.20) |  |  | 2.04 (1.21, 3.44) |  | 1.49 (0.88, 2.51) |
| **Small for gestational age** |  |  |  |  |  |  |
| No | Ref. |  |  | Ref. |  |  |
| Yes | 1.40 (0.91, 2.13) |  |  | 1.55 (0.93, 2.58) |  |  |
| **Smoking during pregnancy** |  |  |  |  |  |  |
| No | Ref. |  |  | Ref. |  | Ref. |
| Yes | 1.69 (1.21, 2.35) |  |  | 1.69 (1.21, 2.37) |  | 1.4 (0.99, 1.96) |
| HR, Hazard ratio; CI, confidence interval; Ref., reference  ^1^ N=44,941, including 164 suicide cases.  ^2^ N=49,714, including 175 suicide cases.  ^3^ N=48,193, including 172 suicide cases.  ^4^ N=48,467, including 174 suicide cases.  ^5^ N=48,136, including 173 suicide cases. | | | | | | |

**Table S4. Results of survival analyses of the Unadjusted Associations of Sociodemographic Factors, Pregnancy, Birth and Neonatal Complication Scores, and other Individual Risk Factors with Suicide Mortality by Sex and Sex interactions.**

|  | **Unadjusted HR (95%CI)**  **Men, 214 suicides** | **Unadjusted HR (95%CI)**  **Women, 74 suicides** |
| --- | --- | --- |
| ***SOCIODEMOGRAPHIC FACTORS*** |  |  |
| **Race** |  |  |
| White | Ref. | Ref. |
| Black | 0.66 (0.5, 0.88) | 0.54 (0.33, 0.87) |
| Other | 0.4 (0.19, 0.81) | 0.13 (0.02, 0.95) |
| Interaction | LR χ^2^(2) =1.73, *p*=0.422 |  |
| **Family structure** |  |  |
| Both parents at home | Ref. | Ref. |
| Single or Married & Father absent | 0.74 (0.48, 1.15) | 0.84 (0.42, 1.71) |
| Sep/Widowed/Divorced & Father absent | 1.18 (0.75, 1.86) | 1.66 (0.84, 3.25) |
| Interaction | LR χ^2^(2) =0.68, *p*=0.711 |  |
| **Household density** |  |  |
| < 1 persons per bedroom | Ref. | Ref. |
| 1-<1.5 persons per bedroom | 1.50 (1.06, 2.11) | 0.95 (0.47, 1.94) |
| ≥1.5 persons per bedroom | 0.95 (0.70, 1.30) | 1.36 (0.83, 2.24) |
| Interaction | LR χ^2^(2) =4.32, *p*=0.115 |  |
| **Poverty ratio category** |  |  |
| 90^th^ percentile | Ref. | Ref. |
| 75^th^ percentile | 1.40 (0.80, 2.42) | 1.62 (0.57, 4.61) |
| 50^th^ percentile | 1.10 (0.65, 1.87) | 1.16 (0.42, 3.22) |
| 25^th^ percentile | 1.33 (0.78, 2.25) | 1.39 (0.50, 3.82) |
| 10^th^ percentile | 1.25 (0.70, 2.24) | 2.37 (0.87, 6.47) |
| <10^th^ percentile | 0.89 (0.47, 1.69) | 1.01 (0.31, 3.30) |
| Interaction | LR χ^2^(5) =2.60, *p*=0.761 |  |
| **Parental education** ≤ **12 years** |  |  |
| No | Ref. | Ref. |
| Yes | 2.17 (1.29, 3.68) | 2.68 (0.98, 7.34) |
| Interaction | LR χ^2^(1) =0.13, *p*=0.715 |  |
| **Parental occupation at birth** |  |  |
| Non-manual | Ref. | Ref. |
| None/student | 1.08 (0.58, 1.99) | 1.02 (0.35, 2.96) |
| Manual | 1.26 (0.94, 1.68) | 1.35 (0.82, 2.25) |
| Interaction | LR χ^2^(2) =0.09, *p*=0.955 |  |
| **Birth order** |  |  |
| 1^st^ | Ref. | Ref. |
| 2^nd^ | 1.27 (0.84, 1.92) | 1.30 (0.59, 2.90) |
| 3^rd^ | 1.34 (0.86, 2.09) | 2.46 (1.17, 5.14) |
| ≥4^th^ | 1.53 (1.07, 2.18) | 2.00 (1.02, 3.90) |
| Interaction | LR χ^2^(3) =2.47, *p*=0.480 |  |
| ***COMPLICATION SCORES*** |  |  |
| **Pregnancy complication score** |  |  |
| 0 | Ref. | Ref. |
| 3-4 | 1.79 (0.70, 4.53) | 1.45 (0.43, 4.90) |
| 5-7 | 2.42 (0.97, 6.02) | 1.18 (0.35, 4.00) |
| 8-10 | 2.70 (1.07, 6.78) | 1.70 (0.50, 5.76) |
| >10 | 2.62 (1.05, 6.53) | 1.18 (0.35, 4.07) |
| Interaction | LR χ^2^(4) =3.26, *p*=0.516 |  |
| **Labor and Delivery complication score** |  |  |
| 0 | Ref. | Ref. |
| 3-4 | 1.34 (0.94, 1.91) | 0.81 (0.45, 1.44) |
| 5-7 | 1.54 (1.01, 2.34) | 0.63 (0.29, 1.41) |
| >7 | 1.01 (0.68, 1.50) | 0.94 (0.52, 1.70) |
| Interaction | LR χ^2^(3) =5.47, *p*=0.141 |  |
| **Neonatal complication score** |  |  |
| 0 | Ref. | Ref. |
| 3-4 | 0.76 (0.53, 1.07) | 1.08 (0.57, 2.01) |
| 5-8 | 0.71 (0.48, 1.05) | 1.22 (0.63, 2.36) |
| >8 | 0.77 (0.53, 1.14) | 1.21 (0.61, 2.40) |
| Interaction | LR χ^2^(3) =2.26, *p*=0.5.21 |  |
| ***INDIVIDUAL RISK FACTORS***  ***(Included in complication scores)*** |  |  |
| **Mother age, years** |  |  |
| <20 | 1.10 (0.76, 1.60) | 1.12 (0.64, 1.96) |
| 20-24 | Ref. | Ref. |
| 25-29 | 1.30 (0.90, 1.87) | 0.71 (0.36, 1.39) |
| 30-34 | 1.32 (0.84, 2.06) | 0.87 (0.40, 1.89) |
| ≥35 | 1.63 (1.01, 2.64) | 0.68 (0.24, 1.94) |
| Interaction | LR χ^2^(4) =5.16, *p*=0.271 |  |
| **Maternal psychiatric history** |  |  |
| No | Ref. | Ref. |
| Yes | 1.44 (0.94, 2.20) | 1.49 (0.74, 3.00) |
| Interaction | LR χ^2^(1) =0.01, *p*=0.932 |  |
| **Birth weight** |  |  |
| <1,500g | 1.87 (0.46, 7.52) | - |
| 1,500-2,499g | 1.13 (0.70, 1.81) | 1.22 (0.63, 2.38) |
| 2,500-4,000g | Ref. | Ref. |
| >4,000g | 1.41 (0.88, 2.27) | 0.76 (0.18, 3.09) |
| Interaction | LR χ^2^(3) =2.16, *p*=0.340 |  |
| **Small for gestational age** |  |  |
| No | Ref. | Ref. |
| Yes | 1.16 (0.73, 1.84) | 1.43 (0.79, 2.61) |
| Interaction | LR χ^2^(1) =0.30, *p*=0.586 |  |
| **Smoking during pregnancy** |  |  |
| No | Ref. | Ref. |
| Yes | 1.56 (1.16, 2.10) | 1.34 (0.82, 2.17) |
| Interaction | LR χ^2^(1) =0.28, *p*=0.598 |  |
| HR, Hazard ratio; CI, confidence interval; Ref., reference | | |

**Table S5. Results of survival analyses of the Associations of Sociodemographic Factors, Pregnancy, Birth and Neonatal Complication Scores, and other Individual Risk Factors with Suicide Mortality occurring through age 30 and after age 30.**

|  | **Unadjusted**  **(n=288 suicides)**  **HR (95%CI)** | **Death by suicide through 30 years old (n=82 suicides)**  **HR (95%CI)** | **Death by suicide after 30 years old (n=206 suicides)**  **HR (95%CI)** |
| --- | --- | --- | --- |
| ***SOCIODEMOGRAPHIC FACTORS*** |  |  |  |
| ***Race*** |  |  |  |
| White | Ref. | Ref. | Ref. |
| Black | 0.61 (0.48, 0.78) | 0.55 (0.34, 0.87) | 0.65 (0.49, 0.86) |
| Other | 0.32 (0.16, 0.63) | 0.36 (0.11, 1.16) | 0.31 (0.14, 0.70) |
| **Sex of the child** |  |  |  |
| Male | Ref. | Ref. | Ref. |
| Female | 0.35 (0.27, 0.45) | 0.35 (0.21, 0.57) | 0.35 (0.25, 0.47) |
| **Family structure** |  |  |  |
| Both parents at home | Ref. | Ref. | Ref. |
| Single or Married & Father absent | 0.75 (0.52, 1.09) | 0.52 (0.23, 1.21) | 0.84 (0.56, 1.28) |
| Sep/Widowed/Divorced & Father absent | 1.28 (0.88, 1.86) | 2.63 (1.53, 4.51) | 0.79 (0.46, 1.36) |
| **Household density** |  |  |  |
| < 1 persons per bedroom | Ref. | Ref. | Ref. |
| 1-<1.5 persons per bedroom | 1.37 (1.01, 1.86) | 0.98 (0.51, 1.89) | 1.51 (1.07, 2.15) |
| ≥1.5 persons per bedroom | 1.05 (0.81, 1.37) | 1.32 (0.83, 2.11) | 0.95 (0.69, 1.30) |
| **Poverty ratio category** |  |  |  |
| 90^th^ percentile | Ref. | Ref. | Ref. |
| 75^th^ percentile | 1.43 (0.88, 2.33) | 1.98 (0.71, 5.49) | 1.28 (0.73, 2.23) |
| 50^th^ percentile | 1.11 (0.69, 1.77) | 1.63 (0.61, 4.36) | 0.97 (0.57, 1.65) |
| 25^th^ percentile | 1.31 (0.82, 2.09) | 1.51 (0.55, 4.16) | 1.24 (0.73, 2.10) |
| 10^th^ percentile | 1.46 (0.89, 2.40) | 2.86 (1.06, 7.71) | 1.10 (0.61, 1.97) |
| <10^th^ percentile | 0.90 (0.51, 1.58) | 1.23 (0.39, 3.89) | 0.81 (0.43, 1.55) |
| **Parental education** ≤ **12 years** |  |  |  |
| No | Ref. | Ref. | Ref. |
| Yes | 2.25 (1.41, 3.59) | 4.22 (1.33, 13.38) | 1.88 (1.13, 3.13) |
| **Parental occupation at birth** |  |  |  |
| Non-manual | Ref. | Ref. | Ref. |
| None/student | 1.04 (0.61, 1.77) | 0.44 (0.11, 1.87) | 1.29 (0.73, 2.30) |
| Manual | 1.27 (0.99, 1.64) | 1.34 (0.84, 2.13) | 1.25 (0.93, 1.69) |
| **Birth order** |  |  |  |
| 1^st^ | Ref. | Ref. | Ref. |
| 2^nd^ | 1.28 (0.89, 1.84) | 1.50 (0.73, 3.06) | 1.21 (0.79, 1.85) |
| 3^rd^ | 1.56 (1.07, 2.27) | 1.28 (0.57, 2.88) | 1.65 (1.08, 2.52) |
| ≥4^th^ | 1.61 (1.18, 2.21) | 2.27 (1.23, 4.17) | 1.41 (0.97, 2.04) |
| ***COMPLICATION SCORES*** |  |  |  |
| **Pregnancy complication score** |  |  |  |
| 0 | Ref. | Ref. | Ref. |
| 3-4 | 1.66 (0.79, 3.48) | 3.66 (0.48, 27.57) | 1.38 (0.62, 3.06) |
| 5-7 | 1.97 (0.96, 4.08) | 5.13 (0.70, 37.76) | 1.53 (0.70, 3.36) |
| 8-10 | 2.31 (1.11, 4.82) | 6.06 (0.82, 44.96) | 1.78 (0.80, 3.97) |
| >10 | 2.08 (1.00, 4.31) | 3.38 (0.45, 25.48) | 1.93 (0.88, 4.22) |
| **Labor / Delivery complication score** |  |  |  |
| 0 | Ref. | Ref. | Ref. |
| 3-4 | 1.18 (0.87, 1.59) | 1.06 (0.61, 1.83) | 1.24 (0.87, 1.77) |
| 5-7 | 1.22 (0.84, 1.76) | 0.92 (0.45, 1.89) | 1.36 (0.88, 2.08) |
| >7 | 0.99 (0.71, 1.38) | 0.87 (0.47, 1.59) | 1.06 (0.71, 1.57) |
| **Neonatal complication score** |  |  |  |
| 0 | Ref. | Ref. |  |
| 3-4 | 0.86 (0.63, 1.17) | 1.33 (0.74, 2.40) | 0.73 (0.51, 1.04) |
| 5-8 | 0.84 (0.60, 1.18) | 1.07 (0.55, 2.10) | 0.78 (0.53, 1.15) |
| >8 | 0.91 (0.65, 1.28) | 1.14 (0.58, 2.23) | 0.85 (0.58, 1.26) |
| ***INDIVIDUAL RISK FACTORS***  ***(Included in complication scores)*** |  |  |  |
| **Mother age, years** |  |  |  |
| <20 | 1.10 (0.81, 1.51) | 1.00 (0.55, 1.81) | 1.15 (0.80, 1.65) |
| 20-24 | Ref. | Ref. | Ref. |
| 25-29 | 1.13 (0.82, 1.55) | 1.23 (0.69, 2.19) | 1.08 (0.74, 1.59) |
| 30-34 | 1.17 (0.79, 1.72) | 0.81 (0.35, 1.86) | 1.31 (0.84, 2.03) |
| ≥35 | 1.34 (0.87, 2.07) | 1.61 (0.76, 3.41) | 1.23 (0.72, 2.09) |
| **Maternal psychiatric history** |  |  |  |
| No | Ref. | Ref. | Ref. |
| Yes | 1.44 (1.00, 2.06) | 1.15 (0.55, 2.39) | 1.57 (1.03, 2.39) |
| **Birthweight** |  |  |  |
| <1,500g | 1.32 (0.33, 5.31) | - | 1.82 (0.45, 7.35) |
| 1,500-2,499g | 1.07 (0.73, 1.58) | 1.36 (0.70, 2.65) | 0.97 (0.60, 1.55) |
| 2,500-4,000g | Ref. | Ref. | Ref. |
| >4,000g | 1.48 (0.95, 2.32) | 2.10 (1.01, 4.37) | 1.25 (0.71, 2.20) |
| **Small for gestational age** |  |  |  |
| No | Ref. | Ref. | Ref. |
| Yes | 1.09 (0.76, 1.57) | 1.57 (0.87, 2.84) | 0.91 (0.57, 1.44) |
| **Smoking during pregnancy** |  |  |  |
| No | Ref. | Ref. | Ref. |
| Yes | 1.50 (1.16, 1.93) | 1.55 (0.96, 2.49) | 1.48 (1.10, 1.99) |
| HR, Hazard ratio; CI, confidence interval; Ref., reference | | | |
